# Association Between Oral Dysbiosis and Depression: A Systematic Review

**DOI:** 10.3390/jcm14145162

**Published:** 2025-07-21

**Authors:** Paula García-Rios, Miguel R. Pecci-Lloret, María Pilar Pecci-Lloret, Laura Murcia-Flores, Nuria Pérez-Guzmán

**Affiliations:** 1 Gerodontologý an Special Care Dentistry Unit, Morales Meseguer Hospital, Faculty of Medicine, University of Murcia, IMIB-Arrixaca, 30008 Murcia, Spain; paula.garciar@um.es (P.G.-R.); mariapilar.pecci@um.es (M.P.P.-L.); nuria.perez5@um.es (N.P.-G.); 2 Department of Health Sciences, Catholic University San Antonio of Murcia, 30107 Murcia, Spain; lmurcia@ucam.edu

**Keywords:** dysbiosis, oral microbiota, depression, depressive disorder

## Abstract

**Background**: Depression is a mental disorder characterized by a combination of somatic and cognitive disturbances, in which a predominantly sad or irritable mood significantly interferes with the patient’s functioning. This condition can affect individuals of all ages and socioeconomic backgrounds. Currently, various studies are exploring a possible association between oral dysbiosis and depression—an increasingly relevant topic, as confirmation of such a relationship could position the oral microbiota as a potential etiological or diagnostic factor for depression, given its accessibility and ease of analysis. **Aim**: To present a qualitative synthesis of studies addressing how oral dysbiosis influences the onset of depression, as well as the importance of controlling this alteration of the oral microbiota to aid in the prevention of the disease. **Materials and Methods**: The PRISMA guidelines (Preferred Reporting Items for Systematic Reviews and Meta-Analyses) outline the procedures to be followed for conducting this systematic review. The article search was carried out on 22 May 2025, across the PubMed, Scopus, Scielo, and The Cochrane Library databases, using terms related to “depression” and “oral dysbiosis”. Studies published within the last 10 years that addressed the potential association between oral dysbiosis, and depression were included. Furthermore, the quality of the studies was assessed using various tools depending on their design: the Newcastle–Ottawa Scale (NOS) was applied to case-control and cohort studies; the Joanna Briggs Institute (JBI) critical appraisal checklist was used for cross-sectional studies; and experimental studies were evaluated using SYRCLE’s Risk of Bias Tool. **Results**: A total of eleven studies were included in this systematic review. The findings suggest the presence of alterations in the oral microbiota of patients with depression, particularly in terms of composition, structure, and diversity. A reduction in alpha diversity—an indicator of local microbial balance—was observed, along with an increase in beta diversity, indicating greater inter-individual variability, which may be associated with inflammatory processes or immunological dysfunctions. Some studies reported differing results, which may be attributable to methodological variability regarding study design, or the populations sampled. **Conclusions**: This systematic review suggests that the oral microbiome could be considered a diagnostic biomarker and therapeutic target for depression, as the analyzed studies demonstrate a significant association between oral microbiome dysbiosis and this mental disorder. However, the methodological heterogeneity among the studies highlights the need for further research to confirm this potential relationship.

## 1. Introduction

Depression is a common and complex condition characterized by a sad or irritable mood, which is frequently accompanied by somatic and cognitive changes that significantly interfere with the functional capacity of the individual in question, potentially affecting people of all ages and social statuses. In fact, the World Health Organization ranks this condition as the third leading cause of disability worldwide. Regarding its etiology, there is still no definitive answer, but it is believed that psychological, social, and biological processes all contribute to its development. Non-psychotic disorders such as depression and anxiety are only modestly heritable and, in fact, both conditions largely overlap and coexist [1,2]. Nevertheless, certain risk factors been identified that predispose individuals to suffer from this illness, such as female sex, advanced age, physical morbidity, cognitive and functional decline, and bereavement [3,4]. The diagnosis of depression is based on a clinical evaluation carried out by a professional, in which, in addition to the clinical interview, a series of questionnaires are considered, such as the one presented in the Diagnostic and Statistical Manual of Mental Disorders, Fifth Edition (DSM-5). This manual establishes that at least five of the nine symptoms listed must be met for a minimum of 2 weeks, one of which must be either a depressed mood or a loss of interest. On the other hand, using tools such as the Patient Health Questionnaire (PHQ-9), not only the presence but also the severity and progression of the disorder are assessed. If all this were not sufficient, there are also various scales used to evaluate depressive symptoms, among which we highlight the Comprehensive Psychopathological Rating Scale (CPRS) and the Montgomery–Åsberg Depression Rating Scale (MADRS) [4,5,6]. Thus, taking all of this into consideration, depression can be classified as mild, moderate, or severe [4]. The treatment of this mental illness aims to reduce or alleviate symptoms, improve or restore functioning, and enhance patients’ quality of life [1]. For this reason, psychotherapy is considered a suitable treatment option, whether used alone or in combination with antidepressant medications [3]. A recent study reports on the iatrogenic effect of pharmacological treatment, as it is believed to potentially increase the risk of patient relapses, which would imply the creation of a cohort of individuals who do not require medication indefinitely but are unable to discontinue it without relapsing [2].

Finally, as previously mentioned, this mental disorder can affect individuals of different ages and social statuses, with particular emphasis on its presence in specific population groups such as adolescents and pregnant women. In the former, diagnosis is carried out using an adapted version of the Patient Health Questionnaire-9 (PHQ-9), known as the PHQ-A, while in the latter, diagnosis is made using the Edinburgh Postnatal Depression Scale in addition to the PHQ-9 [5,6]. The presence of depression in pregnant women is a controversial issue, as this condition may influence fetal development, birth outcomes, and the development of the baby and child. However, treatment with antidepressants may entail a slight increase in the risk of miscarriage, congenital heart defects, preterm birth, and transient neonatal symptoms. Nevertheless, the effects resulting from exposure to antidepressants require further consistency, due to the difficulty in disentangling the contribution of the disorder from the effect of the medication itself. For all these reasons, non-pharmacological strategies, such as interpersonal psychotherapy, are preferred during pregnancy [5].

This systematic review aims to study the association between depression and the oral microbiota; therefore, it is important to provide information about this group of bacteria. The oral microbiota consists of specific, complex microbial ecosystems whose main characteristic is their dynamism, as they vary from one individual to another due to external factors such as diet, tobacco use, or hygiene habits. This oral microbiome contributes to the maintenance of human health, unless it undergoes alterations (dysbiosis), which may influence the development of diseases. As a result, the oral microbiota is involved in both oral diseases and diseases affecting distant organs. Hence, the importance is on maintaining a healthy microbial balance, which must be achieved and preserved during the developmental period of the oral microbiota, since once it reaches maturity, it becomes so stable that it is difficult to modify. In fact, the period between 4 and 18 months of age would be the most accessible time to control microbial balance, especially through dietary adjustments, as by that time the foundation of the adult oral microbiota has not yet been established [7].

As previously stated, the oral microbiota can contribute to the development of diseases in distant organs, among which neurological disorders are particularly noteworthy. Brain function impairment may result from the transfer of oral bacteria to the brain via the trigeminal nerve and the olfactory system, which connect the oral cavity to the olfactory bulb, or through the entry of oral bacteria into the bloodstream, by which these microorganisms reach the brain directly and disrupt the blood–brain barrier due to the inflammatory response they generate. Therefore, the oral–gut–brain axis has gained increasing relevance, as good oral hygiene and diet could lead to improvements in the symptomatology of patients with neurological disorders [8].

The possible association between oral dysbiosis and depression is a topic of great relevance, as the existence of such a link could establish oral dysbiosis as an etiological or diagnostic factor for depression due to its easy accessibility and study. Therefore, its management could help prevent the onset of this condition.

The aim of this systematic review was to present a qualitative synthesis of studies addressing how oral dysbiosis influences the onset of depression, as well as the importance of controlling this alteration of the oral microbiota to aid in the prevention of said disease.

## 2. Materials and Methods

The PRISMA 2020 guideline, an acronym for “Preferred Reporting Items for Systematic Reviews and Meta-Analyses” [9], was the one considered for the execution of this systematic review. In addition, the review was registered in the PROSPERO database (International Prospective Register of Systematic Reviews) under registration number CRD420251059309.

A series of inclusion and exclusion criteria were followed to carry out this systematic review. Therefore, articles published between 2015 and 2025 were included, as well as those reporting information on the relationship between the presence of oral dysbiosis and depression.

On the other hand, studies linking the presence of depression with other aspects or those in which the patients studied did not have this pathology were not admitted. Furthermore, articles that did not meet the inclusion criteria were also excluded.

To establish the inclusion criteria, the PICO model was followed: population/problem (P): patients with depression; intervention (I): presence of oral dysbiosis; comparison/control (C): patients without depression; outcome (O): association of the presence of oral dysbiosis with depression. Hence, the resulting PICO question would be is there an association or causal relationship between oral dysbiosis and the development of depression?

### 2.1. Search Strategy

PubMed, SciELO, The Cochrane Library, and Scopus were the databases selected to conduct comprehensive search for articles. This research was carried out on 22 May 2025, and included studies that provided clarification on the possible association between oral dysbiosis and depression.

The MeSH (Medical Subject Headings) thesaurus was used to obtain the necessary search terms. The terms related to “oral dysbiosis” were: “dysbiosis”, “microbial imbalance”, “oral microbiota”, “oral microbiome”, and “oral bacteria”. In contrast, the terms related to “depression” were “depression”, “depressive disorder”, “major depressive disorder”, and “mood disorders”. The terms were combined using the Boolean operators “AND” and “OR.” The following Table 1 shows the data resulting from the search for studies in the databases.

### 2.2. Study Selection

Following this procedure, the studies obtained from the search were imported into the EndNote reference manager, and duplicates were removed. Subsequently, a first selection process was carried out by reading the title and abstract of each article, excluding studies that did not meet the inclusion criteria. Finally, the selected documents were read in full to determine their eligibility.

### 2.3. Data Extraction

To conduct a comprehensive analysis of the selected studies, the following categories were taken into consideration for each of them: the author and year of publication, the type of study included, the number and age of participants, characteristics of the oral microbiota analyzed, the presence or absence of a comparative analysis establishing an association between patients with oral dysbiosis and depression, and the conclusions drawn from the study.

### 2.4. Quality Analysis

One experimental study, one cohort study, two case-control studies, and seven cross-sectional studies constitute this systematic review. To conduct the quality assessment of these studies, the following tools were employed: the Newcastle–Ottawa Scale (NOS) [10], the Joanna Briggs Institute (JBI) [11] Critical Appraisal Tool, and SYRCLE’s Risk of Bias Tool [12]. The first assesses the quality of case-control and cohort studies, the second evaluates cross-sectional studies, and the third is used to estimate the risk of bias in experimental studies. The Newcastle–Ottawa Scale [10] examines three specific domains for each study type and, depending on the fulfillment of the criteria established for each domain, awards “stars”. The domains evaluated for case-control studies are selection, comparability, and exposure, whereas for cohort studies, the exposure domain is replaced by outcome. Only one star is awarded per domain, except for comparability, which can receive a maximum of two stars. According to this tool, studies that received between seven and nine stars were classified as low risk of bias; those with four to six stars were considered to have a moderate risk of bias; and those with zero to three stars were considered high risk of bias. On the other hand, the JBI Critical Appraisal Tool [11] was used to assess the quality of cross-sectional studies, as it outlines the type of information these studies should include according to its recommendations. To determine the methodological quality of a cross-sectional study, eight criteria established by this tool were followed. These criteria help identify the presence of bias in the design, conduct, and analysis of the study. Each criterion was rated as “yes”, “no”, “unclear”, or “not applicable”. Based on this, studies were considered to have a low risk of bias if they met between six and eight criteria, a moderate risk if they met between four and five criteria, and a high risk of bias if they met between zero and three criteria. Finally, SYRCLE’s Risk of Bias Tool [12] (developed by the Systematic Review Centre for Laboratory Animal Experimentation) was used to assess the risk of bias in experimental studies involving animals. This tool analyzes selection bias, performance bias, detection bias, attrition bias, reporting bias, among others, based on compliance with 10 items organized into six domains. Each item is rated as “yes”, “no”, or “unclear”. Studies were classified as having a low risk of bias if they fulfilled seven or more items and presented no “no” responses or only one; as moderate risk if they fulfilled between four and six items and had no more than two or three “no” responses; and as high risk if they had three or fewer “yes” responses or four or more items rated as “no”. Studies were required to meet at least half of the established criteria in order to be included in this systematic review. To evaluate the included articles, the following procedure was followed: first, two reviewers (PGR and FJRL) independently analyzed and rated each study, then compared results and identified potential discrepancies. In cases where discrepancies arose, they were resolved by consensus.

## 3. Results

### 3.1. Study Selection and Flow Diagram

**Figure 1:**
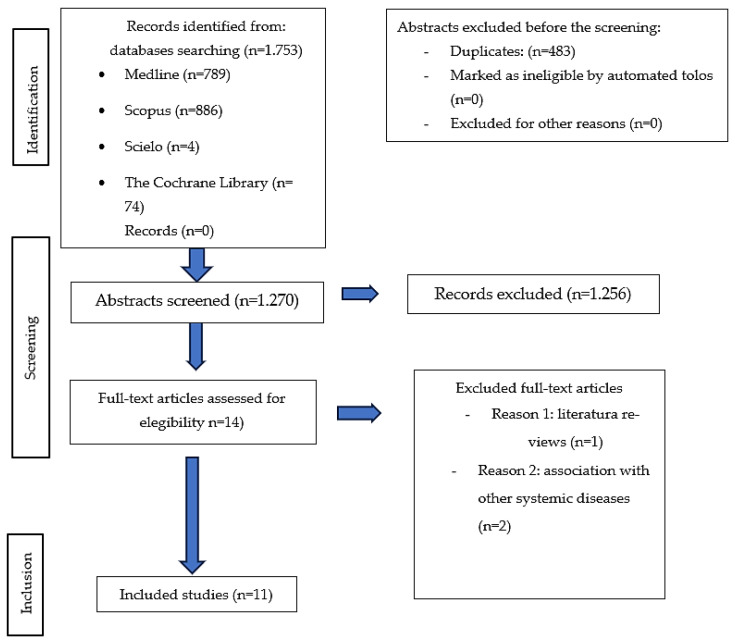
Flow diagram.

The results of the study selection process are presented in Figure 1. As previously mentioned, various databases were used to conduct the literature search, yielding a total of 1753 references. Of these, 789 were retrieved from PubMed, 886 from Scopus, 74 from The Cochrane Library, and 4 from Scielo. Subsequently, 483 duplicate articles were removed using reference management software, resulting in 1270 studies being screened based on title and abstract. Of these, 1256 references were excluded for not meeting the inclusion criteria, as they either associated gut microbiota—rather than oral microbiota—with depression, did not include patients diagnosed with depression, or linked depression with other conditions. Consequently, only 14 articles were selected for full-text review. Of these, three articles were excluded for the following reasons: one was classified as a review article (n = 1), and two included patients with depression who also presented with other systemic conditions (n = 2). In conclusion, 11 articles were selected as they met the previously established inclusion criteria and provided information on the association between oral dysbiosis and depression.

### 3.2. Data Extraction

#### Types of Studies

Two case-control studies, seven cross-sectional studies, one experimental study using animal models and one cohort study were included, as shown in Table 2, Results of the association between oral dysbiosis and depression.

### 3.3. Quality Analysis

**Figure 2:**
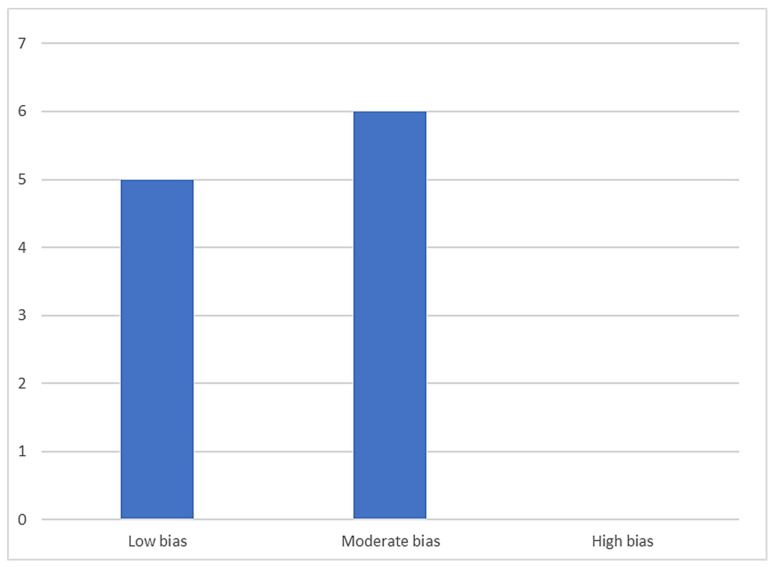
Distribution of studies according to bias.

Table 3, Table 4, Table 5 and Table 6 present the data resulting from the quality analysis conducted using the NOS, SYRCLE, and JBI guidelines, respectively. Of a total of 11 articles included in this systematic review, 6 were assessed as having a moderate risk of bias, and 5 as having a low risk of bias. Conversely, no studies exhibited a high risk of bias. A summary of this is shown in Figure 2.

### 3.4. Bibliometric Analysis

**Figure 3:**
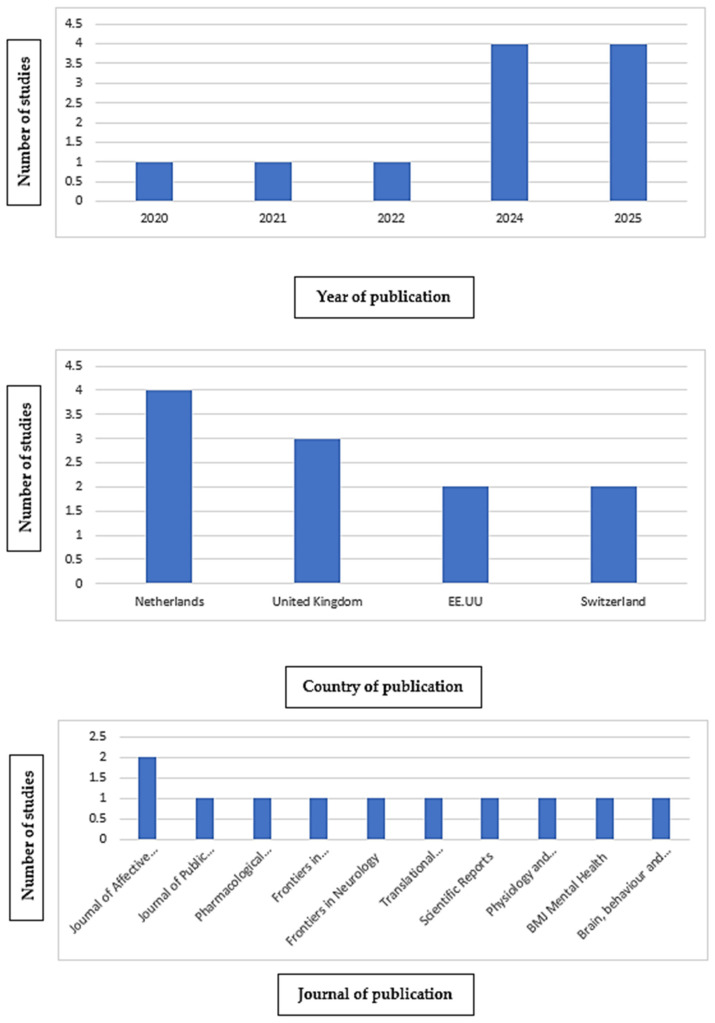
Distribution of the articles included in this review.

Figure 3 illustrates the distribution of the included studies according to publication year, country, and journal of publication. Regarding the year of publication, a progressive increase in studies is observed from 2020 to 2025, except for the year 2023. Up to four articles were published in both 2024 and 2025. Concerning the country of publication, these articles have been disseminated across four different countries, with the Netherlands being the country with the highest number of publications. Finally, there is a wide variety of journals publishing these articles. Each journal contains one study, except for the Journal of Affective Disorders, which includes two publications.

## 4. Discussion

Depression is defined as a mental disorder that affects individuals of all ages and social statuses, potentially impacting on their functional capacity, as it is characterized by inducing somatic and cognitive changes in the affected population—most notably, a persistently sad or irritable mood [1]. Diagnosis is carried out through a clinical evaluation performed by a professional, which considers the clinical interview, and the data obtained from the specific questionnaire used to assess the patient’s mental health [4,5,6]. Currently, psychotherapy is considered a viable therapeutic option for this condition, either alone or in combination with antidepressant pharmacological treatment [3].

This review highlights the notion that changes in the oral microbiota occur in patients experiencing a depressive state, in terms of characteristics, composition, and structure. In fact, based on these findings, a hypothesis has emerged suggesting a possible association between oral dysbiosis and depression. Patients suffering from this mental illness exhibit lower alpha diversity of the oral microbiota compared to healthy individuals—alpha diversity being the tool used to assess how varied and balanced the microbiome is within the oral cavity. However, an increase in beta diversity has been observed in these patients, indicating greater variability in the microbial community among individuals with depression and, consequently, a stronger link to inflammatory processes or immune imbalances [13,14,15]. Nevertheless, methodological variability regarding study design and population samples underscores the need for further research to confirm this association [19].

Moreover, various studies have identified the oral microbiota as a potential contributor to the development of diseases—not only those affecting the oral cavity but also distant organs, with particular emphasis on neurological disorders. Therefore, if the existence of such a link is confirmed, oral dysbiosis could be established as a diagnostic, prognostic, and therapeutic factor for depression, owing to the ease of access and study of the oral environment [8]. Considering this, the present review was conducted with the aim of investigating the characteristics of the oral microbiome in patients with depression, to determine the potential association between these two conditions.

The oral cavity—specifically, the composition and characteristics of the oral microbiome—has gained increasing relevance in the development of mental disorders such as depression. As a result, recent years have seen a growing number of studies investigating the association between these two conditions. In the cross-sectional study conducted by Zheng et al. [13], a sample of 6212 participants from the National Health and Nutrition Examination Survey (NHANES) between 2009 and 2012 was analyzed. Of these, 10.03% were diagnosed with depression using the Patient Health Questionnaire (PHQ-9), and saliva samples were collected from all participants to assess the diversity of their oral microbiome. Based on these data, it was found that alpha diversity was lower in patients with a higher risk of depression, indicating a negative association between alpha diversity and depressive state. Regarding beta diversity, significant differences were observed between individuals with different levels of depression. Similar results were reported in the study by Zhang et al. [14], which used comparable methods to assess mental health and the oral microbiota in a sample of 1497 participants, 111 of whom were diagnosed with depression. This study suggested that variations in beta diversity could influence brain function, affecting neuronal activity through the inflammatory and immune responses they elicit. It also proposed that a higher alpha diversity of the oral microbiota may serve as a protective factor against the development of depression. Other studies have gone further, investigating not only variations in alpha and beta diversity but also the specific bacterial composition of the oral cavity. One such study, conducted by Lou et al. [15], stands out. This research was divided into two parts. The first part analyzed a sample of 157 participants aged 18 to 65, including 70 healthy controls and 87 patients with depression, assessed using the Hamilton Depression Rating Scale (HAMD-17). Saliva samples were collected from all participants for oral microbiome analysis. Significant differences in microbiota composition were found between the healthy and depressive groups, with a notable increase in *Pseudomonas* and *Capnocytophaga*, and a marked decrease in *Streptococcus*, *Leptotrichia*, and *Solobacterium* in the depression group. Additionally, differences in salivary metabolite profiles were observed, particularly in the levels of the omega-3 polyunsaturated fatty acid (EPA), which appeared to exert a protective effect against depression-related behaviors. The second part of the study involved an experimental model using 66 female Kumming mice, divided into healthy controls and a group subjected to chronic restraint stress (CRS) to induce depression-like behaviors. The results showed that CRS altered the structure and composition of the oral microbiota in mice, with changes like those observed in the oral microbiome of patients with depressive symptoms. This animal model was used to support the hypothesis of a bidirectional association between alterations in the oral microbiota and depression.

Several studies have investigated the association between the oral microbiome and depressive symptoms, as well as its relationship with other systemic conditions, including anxiety, sleep duration, and the patient’s periodontal status. In the article published by Li et al. [16], a cohort of 502,656 participants aged 40 to 69 from the UK Biobank (UKB) was recruited. Among them, 154,360 individuals were diagnosed with depression based on PHQ-9 scores, and 157,459 reported self-identified depressive symptoms. The study revealed a significant common interaction between the microbiomes of the tongue dorsum and saliva with the presence of depressive and anxiety symptoms. Specifically, five salivary microbiomes and ten tongue dorsum microbiomes were identified as causal risk factors for depression, including bacterial taxa such as *Actinobacteria* and *Firmicutes*. However, the effects of the oral microbiome on anxiety and depression differed by sex, which may be attributed to gender-related differences in the oral microbiome. For example, the family *Pasteurellaceae* and the genus *Haemophilus* were more abundant in women, while the genus *Capnocytophaga* was significantly more prevalent in men. On the other hand, the study by Liu et al. [17] demonstrated that sleep duration influences the relationship between the oral microbiome and depression. This study used data from the National Health and Nutrition Examination Survey (NHANES) collected between 2009 and 2012, from which 4692 subjects were selected. Sleep quality in individuals over 20 years of age was classified as follows: insufficient sleep (less than 4 h or between 5 and 7 h), adequate sleep (between 7 and 9 h), and excessive sleep (10 h or more). The findings indicated that both insufficient and excessive sleep were associated with higher depression scores compared to adequate sleep. However, insufficient sleep posed a greater risk of depression than excessive sleep. Furthermore, previous findings were confirmed, showing reduced alpha diversity in the salivary microbiome of individuals with higher levels of depression. In this context, alpha diversity moderates the relationship between sleep duration and depression, while beta diversity provided further insight into the interaction among the oral microbiome, sleep duration, and depressive symptoms. Additionally, the study by Malan-Müller et al. [18] explored the connection between the oral microbiome, depression, and the patient’s periodontal condition. This research included two Spanish cohorts: PsicoBioma and TRIAD. Mental health was assessed using the Center for Epidemiologic Studies Depression Scale (CESD), oral microbiota was evaluated through saliva samples, and periodontal status was determined via a periodontal health questionnaire. Among the participants, 148 were classified as depressive subjects, and 81 as individuals with severe periodontitis. The results demonstrated that both periodontal status and mental state influence the composition of the oral microbiota. In fact, individuals with higher levels of depression showed a significant increase in *Capnocytophaga* and *Eggerthia*, bacteria associated with the presence of periodontitis.

As previously mentioned, depression is a mental disorder that does not discriminate based on age or social status. This review includes studies that address the association between the oral microbiome and depression in adolescents and pregnant women. The articles published by Wingfield et al. [19], Simpson et al. [20], and Zeng et al. [21] report findings related to changes observed in adolescent patients. In the first of these studies, a total of 83 participants were analyzed, of whom 40 were diagnosed with depression using an adapted version of the Composite International Diagnostic Interview (CIDI), while the remaining 43 comprised the control group. Saliva samples were collected from all participants to identify potential variations associated with their mental health status. The results revealed that alpha diversity was significantly higher in the depression cohort. Furthermore, the structure of the oral microbiome differed significantly between the two groups. Specifically, 21 bacterial taxa showed differential abundance between depressed participants and healthy controls, of which 4 were bacterial genera and 17 bacterial species. Among the bacteria found to be more abundant in individuals with depression were *Prevotella nigrescens* and *Neisseria*, while 19 other taxa, including *Rothia*, *Treponema*, and *Fusobacteria*, were significantly less abundant in this group. In the study published by Simpson et al. [20], a sample of 66 adolescents aged 14 to 18 years was analyzed 33 with mild depressive symptoms and 33 with severe or high depressive symptoms. Depressive symptoms were assessed using the Center for Epidemiologic Studies Depression Scale (CESD). The characteristics of the oral microbiome were estimated from saliva samples collected from each participant. Adolescents with higher depressive symptoms obtained significantly higher CESD scores compared to the mild-symptom group, which was also associated with greater oral health problems. Regarding microbial diversity, analyses of alpha and beta diversity in the oral microbiome did not reveal significant differences between the depression groups (high vs. low symptoms), nor were significant associations found between these diversity measures and CESD scores. However, differences were observed in bacterial composition between the groups. Specifically, significant positive correlations were identified between depressive symptoms and the abundance of certain bacterial families and genera: the families *Spirochaetaceae* and *Actinomycetaceae*, the Genera *Mitsuokella* and *Bacteroidales*, and the species *Actinomyces* and *Prevotella salivae*. In summary, although no significant associations were found between overall oral microbiome diversity and depressive symptoms in adolescents, specific bacterial taxa were identified as being related to these symptoms. Finally, the study by Zeng et al. [21] investigated a cohort of 74 patients aged 12 to 17, 37 of whom were diagnosed with depression based on DSM-5 criteria, using the Mini International Neuropsychiatric Interview for Children and Adolescents (MINI-KID). Depressive symptoms were additionally assessed using two clinical scales: the 24-item Hamilton Depression Rating Scale (HAMD-24) and the Children’s Depression Inventory (CDI). The diversity of the participants’ oral microbiome was evaluated using diversity indices such as the Abundance-based Coverage Estimator (ACE) and the Chao richness estimator. The results showed that adolescents with depression exhibited lower microbial diversity, with communities dominated by one or a few species. Moreover, the overall bacterial community structure differed significantly between the depression group and the control group. Regarding oral microbiota composition, the genus *Streptococcus* was relatively more abundant in patients with depressive symptoms, while *Prevotella* showed slightly higher abundance in the control group. In summary, 11 bacterial taxa were found to differ in abundance between the groups: *Streptococcus*, *Porphyromonas*, *Neisseria*, *Fusobacterium*, *Alloprevotella*, *Abiotrophia*, *Absconditabacteriales*, *Eubacterium*, *Parvimonas*, *Delftia*, and *Saccharimonas*.

The studies conducted by Alex et al. [22] and Agranyoni et al. [23] have explored the potential association between oral dysbiosis and depression in pregnant women. In the first of these, a sample of 224 pregnant women was analyzed, divided into groups based on the severity of depressive symptoms—mild and severe. Symptoms were assessed using the Edinburgh Postnatal Depression Scale, and the characteristics of the oral microbiome were evaluated by collecting saliva samples through passive drooling. The results showed that species richness was significantly higher in pregnant women with more severe depressive symptoms, whereas beta diversity did not differ significantly between groups. Furthermore, alterations were observed in the abundance of specific oral bacteria: the phyla *Firmicutes* and *Spirochaetes* were more abundant among participants with more severe depressive symptoms. In the study by Agranyoni et al. [23], a sample of 400 women was included, from whom saliva samples were collected during pregnancy, and mental health questionnaires were completed. Of these, 46 were diagnosed with depression. Depressive symptoms were measured using the Center for Epidemiologic Studies Depression Scale (CES-D). The study aimed to determine whether women with clinically significant depressive symptoms exhibited variations in the diversity of their oral microbiome compared to those without such symptoms. To assess this, the number of observed taxes, the Inverse Shannon diversity index, and phylogenetic diversity were used. Alpha and beta diversities were similar between the groups. While no significant differences in alpha or beta diversity were observed between women with and without clinically significant depressive symptoms, significant differences were identified in taxonomic abundance. The genera *Neisseria*, *Fusobacterium*, *Capnocytophaga*, and *Streptococcus* were found to be less abundant in pregnant women with depression. Moreover, the results differed from those observed in studies conducted on non-pregnant populations, highlighting the potential influence of pregnancy on the relationship between the oral microbiome and mental health.

This systematic review, like the studies included within it, presents several limitations. One notable constraint is the heterogeneity of the study populations: while some investigations analyze large, general cohorts, others focus on specific subgroups such as adolescents or pregnant women. Additionally, there is considerable variability in the methodologies used to characterize the oral microbiome and to clinically assess depression, which may contribute to inconsistencies in the reported findings. Furthermore, most of the included studies are cross-sectional in design, limiting the ability to infer causal relationships. To ensure the review’s relevance and timeliness, only studies published within the past decade were considered, and the selection was restricted to articles written in English or Spanish.

## 5. Conclusions

The findings obtained from this systematic review suggest that the oral microbiome could be considered both a diagnostic biomarker and a therapeutic target for depression, as the research conducted on the reviewed articles reveals a significant association between oral microbiome dysbiosis and the presence of depressive symptoms. Among the proposed mechanisms that could explain this relationship are systemic inflammation and the translocation of bacteria or their metabolites—products of oral dysbiosis—which affect the oral–gut–brain axis. Some studies have shown that lower alpha diversity of the oral microbiota is associated with a higher risk of depression, while beta diversity also differs significantly between depressed and healthy individuals. Moreover, several studies have identified specific bacterial taxa, such as *Streptococcus*, *Neisseria*, and *Prevotella*, whose abundance is associated with depression. However, some smaller studies have not found these results, which could be due to differences in sample size or study design. It is important to note, however, that most of the studies included in this review are cross-sectional in nature, which limits the ability to establish causal relationships. Therefore, further experimental research with larger sample sizes and more consistent designs is required to confirm and expand upon these findings.

## Figures and Tables

**Table 1:** Search strategy.

Databases	Search Field	Results
Medline (Pubmed)	1 # “dysbiosis”, “microbial imbalance”, “oral microbiota”, “oral microbiome”, “oral bacteria”	29,926
	2 # “depression”, “depressive disorder”, “major depressive disorder”, “modo disorders”	271,422
	**1 # AND 2 #**	**789**
SCOPUS	1 # “dysbiosis”, “microbial imbalance”, “oral microbiota”, “oral microbiome”, “oral bacteria”	44,442
	2 # “depression”, “depressive disorder”, “major depressive disorder”, “modo disorders”	1,013,233
	**1 # AND 2 #**	**886**
Scielo	1 # “dysbiosis”, “microbial imbalance”, “oral microbiota”, “oral microbiome”, “oral bacteria”	17
	2 # “depression”, “depressive disorder”, “major depressive disorder”, “modo disorders”	9707
	**1 # AND 2 #**	**4**
The Cochrane Library	1 # “dysbiosis”, “microbial imbalance”, “oral microbiota”, “oral microbiome”, “oral bacteria”	1996
	2 # “depression”, “depressive disorder”, “major depressive disorder”, “modo disorders”	123,956
	**1 # AND 2 #**	**74**

**Table 2:** Results of the association between oral dysbiosis and depression.

Author and Year	Type of Study	Number of Participants and Comparison	Age	Characteristics of the Oral Microbiota	Conclusions
Zheng et al. (2025) [13]	Cross-sectional study	Included 6212 participants from the National Health and Nutrition Examination Survey (NHANES) 2009–2012; 10.03% were diagnosed with depression. Comparison was conducted.	Between 30 and 60 years and 1205 older than 60.	Patients with depression exhibited lower alpha diversity in the oral microbiome. Significant differences in beta diversity (composition) were observed between depressed and non-depressed participants.	Lower alpha diversity is associated with greater risk and severity of depression. Significant differences in oral microbiome composition were noted. Probiotics are suggested to reverse diversity changes, though causality cannot be confirmed.
Zhang et al. (2025) [14]	Cross-sectional study	A total of 1497 patients from NHANES 2009–2012 were analyzed; 111 had severe depression and 1386 did not. Comparison was conducted.	45 participants were under 30, 606 over 50, and the rest between 30 and 50.	Alpha diversity, measured by observed ASVs, was negatively correlated with PHQ-9 scores and associated with a lower risk of depressive symptoms. Beta diversity showed statistically significant group differences.	Higher alpha diversity of the oral microbiome may be a protective factor against depression. Oral microbiome analysis could assist in early identification and intervention in mental health. Associations are shown, not causality.
Lou et al. (2024) [15]	Experimental study (animals)	157 human patients were recruited (87 with depressive symptoms, 70 healthy controls). Animal experiments included a variable number of mice. Comparison was conducted.	18–65 years	No significant differences were found in alpha diversity, but beta diversity revealed significant differences in oral microbiome structure. Increased abundance of *Pseudomonas* and *Capnocytophaga*, and reduced *Streptococcus*, *Leptotrichia*, and *Solobacterium* in depressed patients.	Oral microbiome dysbiosis and metabolic function may be relevant to depression pathogenesis. Microbial and metabolomic compositions differ significantly between depressed patients and controls.
Li et al. (2022) [16]	Cohort study	Based on GWAS data of oral microbiomes: 2017 tongue dorsum samples and 1915 saliva samples. Genetic variant effects were compared.	50–60 years	Significant interactions between salivary and tongue dorsum microbiomes related to anxiety and depression. *Eggerthia* (saliva) was associated with depression.	Explored the relationship between oral microbiomes, anxiety, and depression. Understanding this link may enhance knowledge of pathogenesis and support the development of diagnostic targets.
Liu et al. (2024) [17]	Cross-sectional study	Analyzed data from 4692 NHANES 2009–2012 participants. Classified by depression stage and alpha/beta diversity quartiles. Comparison was conducted.	20–60 years; 24% were over 60.	Alpha diversity moderates the relationship between sleep duration and depression. Lower alpha diversity intensified depressive effects. Beta diversity was associated with depression scores.	Oral microbiome diversity moderates the relationship between sleep duration and depression risk. Significant associations were found between alpha/beta diversity and depression scores.
Malan-Müller et al. (2024) [18]	Cross-sectional study	Total of 470 participants; 164 were mentally healthy controls. Comparison was conducted.	Mean age: 40 years	Alpha diversity was not affected by depression, but beta diversity was significantly influenced by mental health variables. *Prevotella histicola*, *Lancefieldella*, *Oribacterium asaccharolyticum*, and *Eggerthia* were positively associated with depression scores.	Oral microbiome composition was significantly influenced by mental health and periodontal outcomes. Functional prediction analyses suggest a role for tryptophan metabolism in the oral–brain axis. The study reports microbial associations, not causality.
Wingfield et al. (2021) [19]	Case-control study	Total of 83 participants: 40 with depression and 43 healthy controls. Comparison was conducted.	Mean age: 20 years	No significant alpha diversity differences, but beta diversity varied significantly; 21 bacterial taxa were differentially abundant, including *Prevotella nigrescens* and *Neisseria*, which were more abundant in depressed participants.	The human oral microbiome has potential as a source of novel biomarkers for diagnosis and treatment of depressive disorders. The study opens avenues to investigate microbiome composition changes in depression etiology.
Simpson et al. (2020) [20]	Cross-sectional study	Total of 66 adolescents: 33 with low and 33 with high depressive symptoms. Comparison was conducted.	14–18 years	Alpha and beta diversity did not differ by depression symptoms. However, bacterial taxa such as *Spirochaetes*, *Treponema*, and *Fusobacterium periodonticum* were positively associated with depression symptoms.	Microbiome composition, not diversity, was associated with depressive symptoms in adolescents. Salivary cortisol and CRP may moderate host-microbiome interactions linked to mood.
Zeng et al. (2025) [21]	Case-control study	Total of 74 participants: 37 with major depressive disorder, 37 healthy controls. Comparison was conducted.	12–17 years	Significant differences in both alpha and beta diversity between depressed and non-depressed groups. Specific taxa, such as *Streptococcus*, were associated with depression.	Oral microbiota alpha and beta diversity differ significantly in adolescents with untreated depression. Specific bacterial taxa may serve as potential biomarkers for depression.
Alex et al. (2024) [22]	Cross-sectional study	Total of 224 pregnant women, grouped by high and low depressive symptoms. Comparison was conducted.	18–34 years	Alpha diversity was not significantly associated with depressive symptoms. Beta diversity also showed no significant differences. However, *Firmicutes*, *Spirochaetes*, *Dialister*, and *Eikenella* were more abundant in those with depressive symptoms.	Multiple aspects of the oral microbiome during pregnancy were associated with maternal mental health. Targeting oral microbes could be a future strategy for supporting maternal mental well-being.
Agranyoni et al. (2025) [23]	Cross-sectional study	Total of 400 pregnant women, 46 of whom had depressive symptoms. Comparison was conducted.	18–45 years	Alpha and beta diversity were similar between depressed and non-depressed groups. *Neisseria*, *Fusobacterium*, *Capnocytophaga*, and *Streptococcus* was less abundant in women with depression.	Pregnant women with depressive symptoms may exhibit altered oral microbiota. *Neisseria* may serve as a potential biomarker for depressive symptoms during pregnancy.

**Table 3:** The quality assessment of the studies using the adapted version of NOS for case-control studies.

Case-Control Studies (NOS)	Selection	Comparability	Exposure	Total Score
Wingfield et al. [19]	It met 2 out of the 4 established criteria.	The main confounding factors were controlled.	It met 2 out of the 3 established criteria.	6
Zeng et al. [21]	It met 3 out of the 4 established criteria.	Partial control of confounding factors.	It met 2 out of the 3 established criteria.	6

**Table 4:** The quality assessment of the studies using the adapted version of NOS for cohort studies.

Case-Control Studies (NOS)	Selection	Comparability	Outcome	Total Score
Li et al. [16]	It met 2 out of the 4 established criteria.	The main confounding factors were controlled.	It met 1 out of the 3 established criteria.	5

**Table 5:** SYRCLE’s risk of bias tool.

Study	1. Appropriate Random Allocation	2. Similar Baseline Characteristics	3. Allocation Concealment	4. Blinding of Personal/Care Givers	5. Blinding of Outcome Assessors	6. Incomplete Data Adequately Handled	7. Selective Reporting Avoided	8. Free from Other Biases	9. Funding Without Conflict of Interest	10. Appropriate Experimental Design	Overall Risk
Lou et al. [15]	Unclear	Yes	Unclear	No	No	Yes	Unclear	Yes	Yes	Yes	Moderate

**Table 6:** JBI checklist evaluation.

Article Title	Clear Inclusion Criteria	Subjects and Setting Described	Exposure Measured Validly	Standard Criteria for Condition	Confounding Factors Identified	Strategies to Deal with Confounding	Outcomes Measured Validly	Appropriate Statistical Analysis	Overall Appraisal	%
Zheng et al. [13]	Unclear	Yes	Unclear	Yes	Yes	No	Yes	Yes	Include	62.5
Zhang et al. [14]	Unclear	Yes	Unclear	Yes	Yes	Yes	Unclear	Yes	Included	62.5
Lou et al. [15]	Yes	Yes	Unclear	Yes	Yes	No	Yes	Yes	Included	75
Liu et al. [17]	Yes	Yes	Unclear	Yes	Yes	Yes	Yes	Yes	Included	87.5
Malan-Müller et al. [18]	Yes	Yes	Yes	Yes	Yes	Yes	Yes	Yes	Included	100
Wingfield et al. [19]	Yes	Yes	Yes	Yes	Yes	Yes	Yes	Yes	Included	100
Alex et al. [22]	Yes	Yes	Yes	Yes	Yes	Yes	Yes	Yes	Included	100
Agranyoni et al. [23]	Unclear	No	Yes	Yes	Yes	Yes	Unclear	Yes	Included	62.5

## Data Availability

No new data were created or analyzed in this study.
